# Chloroform and Dentistry

**Published:** 1877-02

**Authors:** 


### ARTICLE V.

### *Chloroform and Dentistry.*

The mere announcement of another death from chloroform has of late become so frequent that, unless some new pathological changes are noted in connection with it, or some important lesson can be learned in regard to the prevention of an accident which is always deplorable, we are apt to pass it over as a simple fact, which may possibly be of some value to the statistician. The association of the accident with the mere extraction of a tooth does not tend to raise it above the level of tame and common place occurrences. In fact, we have become so accustomed to look upon the two circumstances in the relation of cause and effect, that we cease to wonder at it, and our appetite pales at promise of a recital or details.

The recent death, at Rahway, N. J., is however, of a character which may prove of more than ordinary interest in connection with some of the circumstances attending it. At least, it can do no harm to remind ourselves of the oft-repeated warnings concerning the danger of chloroform at a time when such an example of their significances is still fresh.

In another column we present a full account of the case. The manner in which the accident was produced, and the culpable want of care in preventing it, are matters which not only throw the most serious reflections upon the judgment of the dentist, but lay him open to the charge of actual criminality. There can be no doubt that death was due to the use of chloroform in this case; it is equally certain that chloroform should not have been administered at all; and it is also apparent that the person who assumed the responsibility of the administration was thoroughly incompetent.

In regard to the use of chloroform in dentistry there is but one opinion, namely, that it is always dangerous. As a general rule, it should never be administered at all for purposes of tooth extraction. In the present state of professional opinion upon the subject, the dentist who chooses to administer it even in a special case, assumes a responsibility of which he should not be ignorant. So great is the prejudice against this anæsthetic among leading dentists, that many will not allow it to be administered in their offices, even when the direct professional responsibility is assumed by an experienced physician.

Although the fact cannot be very well explained, chloroform has taken more victims from the dentist's chair than from any other place. Indeed, it has gained its reputation as a dangerous article more in connection with simple tooth-drawing than with any other operation, however grave or formidable. A very good reason for the liability to accidents is the erect position of the body of the patient while in the operating chair. Taking this into account, authorities are unanimous in advising that chloroform should never be given except the patient is recumbent.

In the Rahway case the dentist not only defiantly violated the rule never to administer the anæsthetic for tooth-drawing, but he neglected all the usual precautions to guard against an accident. No surgeon cares to assume the responsibility of giving chloroform unless he knows that the stomach of the patient is empty, that the circulatory apparatus is in good condition, and the lungs free from disease.

A previous inquiry into these conditions is as much a part of the administration of any anæsthetic as is the placing of the napkin to the nose. It appears in the case before us that all these preliminaries were neglected. The patient came into the office immediately after having eaten a hearty meal, and, without any question being asked, was at once placed in the operating chair. There was no loosening of waistband or shirt-collar, no examination of the chest—in fact, nothing was done except to order the little fellow t take long and deep inspirations, while the napkin was held closely against the nose. The result could have been easily foreseen. The overwhelming effects of rapid anæsthesia and the crowding impediment of a full stomach, in the most unfavorable of all positions of the body, did not invite death in vain. It would seem that the actual extraction of the tooth was done when the patient was already dead, or, at least, *in articulo mortis.*

The examination of the bodies of patients dying from the effects of chloroform have not thus far given us any satis factory pathological explanation. The lesions have varied with each individual case, and have given rise to as many different theories. The careful and thorough examination of the body of the victim of the Rahway tragedy still leaves the question an open one. From a study of the pathological appearances, and from a personal inspection of all the organs examined, the most reasonable theory which offers itself to our mind is, that death occurred as the result of a direct operation of chloroform upon the nerve-centres, inducing paralysis of the heart. Although there were marked evidences of asphyxia, they were not complete enough to impress those who were present with primary importance in regard to the cause of death. We are aware that in cases of death by paralysis of the heart, both ventricles are usually filled with blood, and that in asphyxia only the right side of the heart is in that condition; but the circumstances of the case may in the absence of what appears to be a better explanation, render the theory of asthenia still reasonably tenable. At the time of death, both cavities of the heart may have been distended, and the subsequent and prolonged application of galvanism to the precordia may have induced a sufficient amount of post-mortem contraction of the heart-muscle to have emptied the cavities. The left ventricle being, for obvious reasons, the one most strongly acted upon, might satisfactorily account for its being entirely empty while the right side still contained some blood. Or, if we wished to carry our theorizing further, we might suppose that both ventricles were entirely emptied after death, and that the dark fluid in the right ventricle was the result of regurgitation. It may be, however, that both asphyxia and asthenia operated together in producing the effect; but the precedence which should be given to either involves the discussion of some questions for which, in the present state of pathology regarding deaths from chloroform, we are not yet prepared.—*Editor Med. Record.*

*The Rahway Chloroform Tragedy.*—The corner's jury brought in the following verdict concerning the recent death by chloroform in Rahway: "We believe that the death of Walter E. Lewis was caused by the administration of chloroform for the purpose of extracting a tooth, and that it was administered by Dr. Warren E. Westlake without a proper examination of the patient; and we consider it gross negligence on the part of the said Dr. Westlake in not making the said examination, and not knowing the nature of the anæsthetic used."—*Med. Record.*

---

